# Effect of the Timing of Amubarvimab/Romlusevimab (BRII-196/198) Administration on Progression to Severe Disease in Elderly Patients with COVID-19 Infection: A Retrospective Cohort Study

**DOI:** 10.1007/s44231-023-00040-9

**Published:** 2023-06-05

**Authors:** Yonghao Xu, Ying Liu, Ruiqiang Zheng, Shujie Si, Yin Xi, Xilong Deng, Gang Wang, Liang Zhou, Manshu Li, Ya Wang, Shuo Zhang, Jianfeng Xie, Xiaoqing Liu, Yi Yang, Xiaoping Tang

**Affiliations:** 1 The First Affiliated Hospital of Guangzhou Medical University, Guangzhou, China; 2 Guangzhou Institute of Respiratory and Health, Guangzhou, China; 3 State Key Laboratory of Respiratory Disease, Guangzhou, China; 4 Guangzhou Eighth People’s Hospital, Guangzhou Medical University, Guangzhou, China; 5 Northern Jiangsu People’s Hospital, Yangzhou, China; 6 The Forth Hospital of Inner Mongolia, Hohhot, China; 7 The People’s Hospital of Dalai Nur District, Manzhouli, China; 8 Zhongda Hospital, School of Medicine, Southeast University, Nanjing, China; 9 Guangzhou Laboratory, Bio-Island, Guangzhou, China

**Keywords:** BRII-196/198, Elderly patients, COVID-19 disease

## Abstract

**Objective:**

Early intervention with neutralizing antibodies is considered to be effective in preventing disease progression in patients with mild to moderate COVID-19 infection. Elderly patients are the most susceptible and at a higher risk of COVID-19 infection. The present study aimed to assess the necessity and possible clinical benefits of the early administration of Amubarvimab/Romlusevimab (BRII-196/198) in the elderly population.

**Methods:**

The present study was designed as a retrospective, multi-center cohort study conducted with 90 COVID-19 patients aged over 60, who were divided into two groups based on the timing of the administration of BRII-196/198 (administration at ≤ 3 days or > 3 days from the onset of infection symptoms).

**Results:**

The ≤ 3 days group exhibited a greater positive effect (HR 5.94, 95% CI, 1.42–24.83; *P* < 0.01), with only 2 patients among 21 patients (9.52%) exhibiting disease progression, compared to the 31 patients among the 69 patients (44.93%) of the > 3 days group who exhibited disease progression. The multivariate Cox regression analysis revealed low flow oxygen support prior to BRII-196/198 administration (HR 3.53, 95% CI 1.42–8.77, *P* < 0.01) and PLT class (HR 3.68, 95% CI 1.37–9.91, *P* < 0.01) as independent predictors of disease progression.

**Conclusions:**

In elderly patients with mild or moderate COVID-19 disease, who do not require oxygen support and had the risk factors for disease progression to severe COVID-19 disease, the administration of BRII-196/198 within 3 days resulted in a beneficial trend in terms of preventing disease progression.

## Introduction

The novel coronavirus (COVID-19) disease caused by the pathogen SARS-CoV-2 led to a deadly pandemic, which led to nearly 53.8 million confirmed cases and 6.3 million deaths worldwide according to the latest data (as of June 22, 2022) on WHO website. Therefore, the exploration and development of effective therapeutic agents for COVID-19 disease emerged as a priority in the research community. Neutralizing antibody-based therapies targeting SARS-CoV-2 provide immediate and passive effective immunization against the COVID-19 disease and are currently considered a powerful supplement to public health measures and preventive vaccination. Over the past two years, seven groups of neutralizing antibody therapies were authorized for emergency use by the FDA or EMA, including Casirivimab/Imdevimab, Bamlanivimab (withdrawn due to lack of efficiency), Bamlanivimab/Etesevimab, Sotrovimab, Regdanvimab, Tixagevimab/Cilgavimab, and Bebtelovimab. Another group named Amubarvimab/Romlusevimab (BRII 196/198) was approved for marketing in China on December 8, 2021 [1–3]. In addition, several other neutralizing antibody-based drugs against COVID-19 have reached phase II/ III clinical trials and are expected to play an important role in the prevention and treatment of COVID-19 disease.

Those elderly population, or those with underlying disease conditions, such as obesity, diabetes, hypertension, or cancer, are tend to be at a higher risk of developing severe COVID-19 disease [4–7]. Among these factors, advanced age is reported as the major risk factor for hospitalization and death. The, the risk of death reportedly increases with age (50–64, 65–74, 75–84, and ≥ 85 years vs. 18–39 years [RRs, 3.11, 5.77, 7.67, and 10.98, respectively]) [5]. Early statistical data from China indicate that the case-fatality rate in elderly patients over 60 is much higher than that of the entire whole population [6]. The latest data from the latest portion of the phase III BLAZE-1 clinical trial [8] also indicates that the majority of COVID-19-related deaths occur in male patients and patients aged 59 years and above. Therefore, it is of great significance to conduct relevant research to explore the therapeutic effect of drugs in the elderly population.

Results of relevant clinical studies have confirmed that the early use of SARS-CoV-2 neutralizing antibodies against SARS-CoV-2 significantly reduces the risk of hospitalization and disease progression in patients with mild and moderate COVID-19 disease [8–16]. Few studies have been conducted among patients with severe or critical illnesses requiring hospitalization, and therapeutic strategies based on neutralizing antibodies for hospitalized patients with severe COVID-19 disease are currently under development.

BRII-196 and BRII-198 are two recombinant human IgG1 monoclonal antibodies, which have been derived directly from human B cells retrieved from patients who had recovered from COVID-19 disease [17]. BRII-196 and BRII-198 target distinct epitope regions in RBD in the coronavirus spike glycoproteins, non-competitively, allowing for the combination to retain neutralizing activity against several SARS-COV-2 variants of concerns [18]. In addition, BRII-196 and BRII-198 have been engineered to include a triple-amino-acid (M252Y/S254T/T256E [YTE]) substitution in the fragment crystallizable (Fc) region, which allows for an extended half-life of the product. According to a previous clinical study, this drug combination is administrated intravenously with BRII-196 1000 mg, followed by BRII-198 1000 mg. BRII Biosciences company has released positive data from the ACTIV-2 phase III trial, which evaluated the efficacy of BRII-196/198 in 837 non-hospitalized COVID-19 patients. A statistically significant reduction of 78% was observed in the composite endpoint of hospitalization and death compared to the placebo group (RR 0.22, 95% CI 0.05–0.86, *P* < 0.01) in the interim analysis [19]. However, the submission regarding the emergency use authorization (EUA) of BRII-196/198 has not been approved by FDA to date. In previous studies, neutralizing antibodies were administered within 10 days of the onset of symptoms [8, 16]. The EUA for other neutralizing antibodies also recommended that the antibodies should be administered as soon as possible, preferably within 7 or 10 days of the onset of symptom [20–23].

In the present report, 28 day results from the present retrospective cohort study, which was conducted for patients aged over 60 years who were treated with BRII-196/198 administration beginning at ≤ 3 days vs. > 3 days, are presented. The study aimed to explore the necessity and possible clinical benefits of neutralizing antibody intervention in the early stage of COVID-19 infection, to provide a reference basis for undertaking real-world treatment decisions regarding COVID-19.

## Materials and Methods

### Study Design and Participants

A retrospective, multi-center cohort study was conducted at the First Affiliated Hospital of Guangzhou Medical University. The study population included the locally infected patients admitted at Guangzhou Eighth People’s Hospital (from May 21st, 2021 to July 10th, 2021), the designated hospital for COVID-19 in Yangzhou (from July 21st, 2021 to August 20th, 2021), and Manzhouli Port Hospital (from November 28th, 2021 to December 18th, 2021). The First Affiliated Hospital of Guangzhou Medical University sent medical teams to the above three hospitals to participate in the management of COVID-19 disease. The study was approved by the Ethics Committees of all three hospitals (ethical approval numbers: 202113200, 2,021,052 and 2,021,001, respectively).

The inclusion criteria were as follows:

- Age above 60 years;
- Hospitalized with a positive diagnosis of COVID-19 disease and a CT value of less than 40;
- Having received an injection of BRII-196/198 monoclonal neutralizing antibody after diagnosis;
- No requirement for respiratory support with high-flow oxygen or above prior to the administration of BRII-196/198.

The exclusion criteria were as follows:

- Having received high-flow nasal oxygen, mechanical ventilation, or extracorporeal membrane oxygenation support prior to the administration of BRII-196/198;
- Having received other monoclonal antibodies or convalescent plasma simultaneously during hospitalization.

Except for receiving a single dose of BRI-196/198, the treatment of the infected patients was referred to the eighth edition of the COVID-19 Diagnosis and Treatment Guidelines [24] issued by the National Health Commission of the People’s Republic of China. Supportive treatment was administered mainly to non-critical patients. Non-critical patients with high-risk factors and those having severe and critical disease conditions were in the prone position. The patients with SpO_2_ below 93% were first provided with oxygen via nasal cannula (according to the severity classification by WHO). However, when the oxygen flow was above 3 L/min, the patients were provided with high-flow nasal oxygen or non-invasive ventilation (NIV) to maintain oxygen saturation above 95%. Critical patients were supported with invasive mechanical ventilation (IMV) and even extracorporeal membrane oxygenation (ECMO).

The use of BRII-196/198 was evaluated and prescribed to patients by clinicians according to the patient’s clinical manifestations combined with high-risk factors (over 60, diabetes, hypertension, chronic lung or kidney disease, immune deficiency, presence of tumor, transplantation, obesity, and heavy smoking history, according to the eighth edition of the COVID-19 Diagnosis and Treatment Guidelines [24]). BRII-196/198 was administrated as a single dose, sequentially, beginning with 1000 mg of BRII 196 followed by 1000 mg of BRII 198, with a saline flush between the two antibodies.

### Data Collection

The data collection was prespecified and included baseline characteristics, risk factors, laboratory test results, and disease prognosis information. This was to reduce the inherent bias. All data collectors were intensive care or respiratory physicians who were trained by the principal investigators of the present study. All collected data were evaluated by two other researchers, while a third researcher assisted in the interpretation of the data when any discrepancies arose. Data collection was performed using uniform standard guidelines and standard data collection tools, with distinct criteria for recording categorical and quantitative variables. All data were collected from the hospital information system of each center.

### Statistical Analysis

The treatment variable was the duration from the onset of symptoms to the time of administration of BRII-196/198 (≤ 3 days or > 3 days). The index date was defined as the date of BRII-196/198 administration.

Baseline characteristics were compared between the patients treated with BRII-196/198 administered at ≤ 3 days vs. those who received BRII-196/198 administration at > 3 days. The comparisons were performed using the *t* test or *Wilcoxon’s rank sum* test for continuous variables and *Pearson’s χ*^*2*^ test or *Fisher’s exact* test for categorical variables, as appropriate. Time to disease progression was plotted as Kaplan–Meier curves and compared using the *log-rank* test. Independent predictors of 28 day COVID-19 disease progression were identified using the Cox proportional hazards model.

A terminal event was defined as the first occurrence of respiratory support requiring high-flow oxygen inhalation and above or death during hospitalization. Disease progression in patients was assessed on an ordinal scale according to the following categories: 1. discharged or ready for discharge; 2. hospitalization in a non-intensive care unit (ICU) without supplemental oxygen; 3. non-ICU hospitalization with supplemental oxygen; 4. ICU or non-ICU hospitalization with non-invasive ventilation or high-flow oxygen; 5. ICU hospitalization with intubation and mechanical ventilation; 6. ICU hospitalization with extracorporeal membrane oxygenation or mechanical ventilation along with additional organ support; 7. death [25]. A grade 4 or higher endpoint was considered a terminal event.

A univariable Cox proportional hazards model was established to evaluate the impact of baseline variables on disease progression in patients. The multivariable Cox proportional hazards model was used for determining the impact of early treatment with BRII-196/198 after adjusting the baseline confounders that were revealed as statistically significant in the univariable model. A* P* value of < 0.05 was considered statistically significant, and all tests were two-tailed. Data analysis was performed using SAS^®^ Software Version 9.4 (SAS Institute Inc., Cary, NC, USA).

## Results

### Demographic and Clinical Characteristics

Among the 167 cases admitted to Guangzhou Eighth People’s Hospital, 23 patients aged over 60 years who had received BRII-196/198 were included in the present study. Among the 235 cases admitted to the designated hospital for COVID-19 in Yangzhou, 54 were included in the present study. Among the 261 cases admitted to Manzhouli Port Hospital, 32 were included in the present study. Among all included patients, 19 cases had used convalescent plasma or other neutralizing antibodies or had received respiratory support above high-flow oxygen therapy prior to drug administration, who, along with 3 cases that had received both, were excluded from the study. Therefore, in total, 90 cases were finally included for statistical analysis in the present study.

The baseline characteristics of the two groups are listed in Table 1. The study population was divided into two groups according to the duration between the onset of symptoms and the time of drug administration (≤ 3 days or > 3 days). Among all included patients, 21 patients had received BRII-196/198 administration within 3 days of symptom onset after COVID-19 infection, while 69 patients had received the drug over 3 days of symptom onset. No significant differences were revealed between the two groups in all characteristics, except for the lymphocyte counts (1.7 × 10^9^/L vs. 1.0 × 10^9^/L; *P* = 0.006). The median age of the study population was 72.5 years (IQR, 68.0–76.0), and 41 patients (45.6% of the total study population) were male. The average BMI of the included patients was 25 kg/m^2^, and almost 72.2% of patients had comorbidity. Only 21 patients among the elderly patients (23.3%) had completed 2 or more doses of vaccine against COVID-19. An average eGFR value of 67.7 mL/min was recorded for 74 patients, while 30 patients among 83 patients (36.1%) had a lymphocyte count below 0.9 × 10^9^/L and 22 patients (26.5%) had platelet (PLT) level below 100 × 10^9^/L. A total of 76 patients presented a median value of 20.0 (IQR, 15.5–23.9) for the Orf and N gene, among which 37 patients (48.7%) had a CT value of less than 20, while 83.6% of the patients tested negative for IgM antibodies and 69.0% for IgG antibodies.

**Table 1:** Comparison of the baseline characteristics of the patients who received BRII-196/198 administration at ≤ 3 days and > 3 days after the onset of symptoms

Characteristics	Total (N = 90)	Days between symptom onset and BRII-196–198 administration		*p* value
		< = 3 Days (N = 21)	> 3 Days (N = 69)	
Age (Year)	72.5 (68.0,76.0)	73.0 (69.0, 75.0)	72.0 (68.0, 77.0)	0.685
Male Sex	41 (45.6%)	13 (61.9%)	28 (40.6%)	0.086
BMI (kg/m^2^)	25.0 ± 3.4	25.1 ± 3.4	25.0 ± 3.4	0.919
BMI > 24	46 (53.5%)	11 (57.9%)	35 (52.2%)	0.663
Comorbidity	65 (72.2%)	15 (71.4%)	50 (72.5%)	0.926
Vaccine status ≥ 2 doses	21 (23.3%)	8 (38.1%)	13 (18.8%)	0.082
Low flow oxygen support before BRII-196–198 administration	31 (34.4%)	4 (19.1%)	27 (39.1%)	0.090
Corticosteroids use	4 (4.4%)	1 (4.8%)	3 (4.4%)	1.000
eGFR (mL/min)	67.7 ± 24.3	66.8 ± 17.1	67.9 ± 25.9	0.875
Lymphocyte (10^9^/L)	1.0 (0.7, 1.6)	1.7 (1.3, 2.0)	1.0 (0.7, 1.3)	0.006
Lymphocyte < 0.9 × 10^9^/L	30 (36.1%)	4 (23.5%)	26 (39.4%)	0.225
PLT (10^9^/L)	127.0 (99.0, 167.0)	125.0 (101.0,167.0)	127.5 (99.0,166.0)	0.928
PLT < 100 × 10^9^/L	22 (26.5%)	4 (23.5%)	18 (27.3%)	1.000
Orf Gene or N Gene ^a^	20.0 (15.5, 23.9)	18.7 (15.6, 23.2)	20.0 (14.6, 23.9)	0.628
CT value < 20 ^b^	37 (48.7%)	10 (62.5%)	27 (45.0%)	0.213
IgM Negative	61 (83.6%)	15 (93.8%)	46 (80.7%)	0.281
IgG Negative	51 (68.9%)	11 (68.8%)	40 (69.0%)	1.000

^a^Orf Gene or N Gene: choose the minimum of the two values

^b^CT value < 20: the value of the Orf gene or N gene is less than 20

### Univariate and Multivariate Analyses

**Fig. 1:**
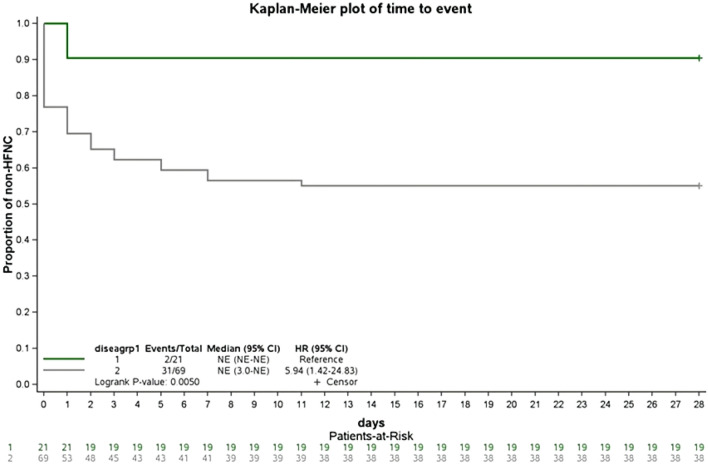
Survival analysis results demonstrating the effect of early drug administration on disease progression

Figure 1 demonstrates the positive effect of the early administration (≤ 3 days) of BRII-196/198 on disease progression (HR 5.94, 95% CI, 1.42–24.83; *P* < 0.01). In the ≤ 3 days group, only 2 patients among 21 patients (9.5%) required respiratory support with high flow oxygen therapy or above, compared to 31 patients among the 69 patients (44.9%) in the > 3 days group who presented disease progression within 28 days of hospitalization. These data indicated that early administration (≤ 3 days) of BRII-196/198 was significant in preventing disease progression compared to the drug administration after 3 days.

Table 2 presents the results of univariate and multivariate Cox regression analyses. The duration between the onset of symptoms and treatment, vaccine status, low flow oxygen support prior to BRII-196–198 administration, lymphocyte class, PLT class, and CT value were revealed to be associated with disease progression in the univariate analysis. The multivariate analysis revealed that whether or not a neutralizing antibody is used within 3 days does not have a significant impact on disease progression (HR 3.81, 95% CI 0.82–17.68, *P* = 0.088). In addition, it revealed low flow oxygen support prior to BRII-196–198 administration (HR 3.53, 95% CI 1.42–8.77, *P* < 0.01) and PLT class (HR 3.68, 95% CI 1.37–9.91, *P* < 0.01) as the independent predictors of disease progression.

**Table 2:** Results of univariate and multivariate Cox regression analyses

Variable	Univariate		Multivariate	
	HR_Crude_ (%95 CI)	*p* value	HR_Adjusted_ (%95 CI)	*p* value
Duration of onset to treatment (> 3 Days)	5.61(1.34–23.48)	0.018	3.81(0.82–17.68)	0.088
Male sex	1.14(0.57–2.25)	0.715		
BMI > 24 kg/m^2^	0.81(0.40–1.63)	0.548		
Comorbidity	1.05(0.49–2.27)	0.895		
Vaccine status < 2 doses	5.57(1.33–23.30)	0.019	3.28(0.41–26.54)	0.265
Low flow oxygen support before BRII-196–198 Administration	2.24(1.13–4.44)	0.021	3.5(1.4–8.8)	0.007
Corticosteroids	2.74(0.83–9.01)	0.097		
Lymphocyte < 0.9 × 10^9^/L	2.67(1.29–5.51)	0.008	1.35(0.56–3.21)	0.503
PLT < 100 × 10^9^/L	2.94(1.43–6.05)	0.003	3.68(1.37–9.91)	0.010
Orf and N Gene > = 20	2.83(1.24–6.44)	0.013	1.95(0.82–4.63)	0.130
IgM Positive	1.97(0.78–4.97)	0.151		
IgG positive	1.27(0.56–2.86)	0.572		

*Variables included in the multivariate analysis are those with statistical significance in univariate analysis, including duration between the onset of symptom to treatment, vaccine status, low flow oxygen support prior to BRII-196–198 administration, lymphocyte class, PLT class, and CT value

## Discussion

The results of the univariate analysis indicated that the duration between the onset of symptoms to BRII-196/198 administration was associated with the clinical outcomes. The ≤ 3 days group exhibited a more positive impact in terms of preventing the disease progression. On the other hand, the multivariate analysis indicated that using neutralizing antibody within 3 days after the onset of symptoms was not a significant independent protective factor (HR 3.81, 95% CI 0.82–17.68, *p* = 0.088), and the hazard ratio was as high as 3.81, suggesting a trend of benefit with early intervention in terms of reducing the risk of disease progression with certain clinical significance. The wide confidential interval could be due to the small sample size of the present study. The final report for Remdesivir applied for the treatment of COVID-19 disease [26] indicated that the patients who receive treatment during the first 10 days after the onset of symptoms have a greater benefit than those who receive treatment beyond 10 days after the onset of symptoms (RR 1.37, 95% CI 1.14–1.64 vs. RR 1.20, 95% CI 0.94–1.52). The comprehensive analysis of the phase II/ III EPIC-HR trial [27] revealed that in patients who received Nirmatrelvir along with Ritonavir within 3 days and 5 days after the onset of symptoms, the relative risk was reduced by 88.9 and 87.8%, respectively. The incubation period of COVID-19 is 1–14 days, and in most cases, it is 3–7 days [24]. The first few days after the onset of symptoms represent a key intervention window to prevent disease progression [28]. Therefore, treatment should be received in the early stage of the disease, which could effectively prevent the progression and even reduce the risk of death due to this disease. The EUA for Bebtelovimab used for the treatment of COVID-19 disease suggests that the administration of antibodies should begin as soon as possible, preferably within 7 days of the onset of symptoms [20]. The EUA for the use of Bamlanivimab, Sotrovimab, and Casirivimab plus Imdevimab recommends administration within 10 days of the onset of symptoms [21–23].

The independent predictors that were associated greatly with disease progression were whether low-flow oxygen was required prior to BRII-196/198 administration and the PLT class, as revealed using the multivariate analysis model. Therefore, the severity of the patient's baseline condition is associated greatly with the clinical outcome. The symptom of dyspnea was reported as a predictor of poor outcomes in elderly patients in a 4 week follow-up study [29]. The results of a multicenter retrospective study involving 1,099 COVID-19 patients [30] indicated that thrombocytopenia was present in 36.2% of the patients during admission to the hospital, and laboratory abnormalities were more prominent in 173 critical/severe patients compared to the non-critical/severe patients. In addition, higher percentages of patients with severe disease required oxygen support therapies. When patients have thrombocytopenia and require low-flow oxygen support, this implies that the patients have a severe baseline status. The joint investigation report on COVID-19 by China and WHO indicated that the duration between the onset of symptoms to progression to severe disease such as hypoxia is usually 1 week [31], although older patients may progress to severe disease and require oxygen support within a further short period after the onset of symptoms due to their poor autoimmune function.

The present study only included the elderly patients who received BRII-196/198, and whether the use of BRII-196/198 conferred additional clinical benefit compared to those who were not administered BRII-196/198 and already required oxygen support at baseline could not be investigated. A real-world study [32] from Singapore, which was conducted during the Delta strain epidemic, reported a 1-month mortality rate of 13.3% (10/75) in COVID-19 patients who were not treated with Sotrovimab, compared to the 5.3% (1/19) of patients in the Sotrovimab group. No clinical death was recorded for any of the 90 patients included in the present study after 1 month of BRII-196/198 administration. These findings indicate that the use of monoclonal antibodies confers certain clinical benefits.

However, so far, no monoclonal neutralizing antibodies have been approved for the treatment of severe COVID-19 disease, and the benefits of monoclonal neutralizing antibodies in patients with severe COVID-19 disease remain debatable. The RECOVERY trial and the ACTIV-3 trail demonstrated that the use of Casirivimab/Imdevimab or Tixagevimab/Cilgavimab could reduce the 90-day mortality rate in patients who did not have a natural antibody response on their own [14, 33]. In the TICO trail [34], BRII-196/198 was observed to have no efficacy over placebo in improving the clinical outcomes in hospitalized adults (median age, 61 [IQR, 50–71] years) with COVID-19 disease. It is noteworthy that the afore-stated trail ended in early termination of participant enrollment because the two antibodies being evaluated could not fulfill the criteria for phase II in the phase III trial, and the final number of patients did not reach the sample size required for the statistical validity test. The subgroup analysis of baseline endogenous neutralizing antibody status indicated that the BRII-196/198 therapy could be effective only in patients who have not yet developed endogenous antibodies. The duration between the onset of symptoms to drug administration for the hospitalization patients in the TICO trail exceeded 12 days, and those patients could have developed endogenous antibodies, which might have contributed to the heterogeneous treatment outcomes observed.

The drugs currently used for the treatment of COVID-19 mainly include neutralizing antibodies, small-molecule drugs, and immunomodulators [2]. The latest COVID-19 treatment guideline by WHO recommends Ritonavir-boosted Nirmatrelvir as the best and the most important treatment for patients with mild and moderate COVID-19 disease who are at the highest risk of hospitalization [35]. SFDA has adjusted the indication of Ritonavir-boosted Nirmatrelvir as “adults with light and common types, within 5 days of the onset of symptoms and with high-risk factors of progression to severe disease” [36]. BRII-196/198, as one of the currently available alternative therapies of Ritonavir-boosted Nirmatrelvir, is a neutralizing antibody developed independently in China. The clinical application and accessibility of this neutralizing antibody are expected to be similar to Ritonavir-boosted Nirmatrelvir in the future, with the commercial launch of the BRII combo on 8th July, 2022. Since this drug has clinical evidence in support of it in the Chinese patient population, it is expected to better reflect the clinical effect in the Chinese population.

Elderly patients are highly susceptible to the novel coronavirus and have a higher risk of disease progression and death [37]. In the present study, 72.2% of the elderly patients had chronic basic diseases, comorbidity, and polypharmacotherapy, which exert serious impacts on the elderly COVID-19 patients [37]. The median eGFR value of these patients was 67.20 (IQR, 49.80–88.00). The liver and kidney function of patients and drug interaction must be considered when using oral anti-viral drugs. Only 21 patients (23.3%) in the present study had completed 2 or more doses of the vaccine against COVID-19. The Cox regression analysis revealed that vaccination is not an independent risk factor for disease progression, suggesting that the early use of monoclonal antibodies could be beneficial for elderly patients regardless of vaccination. With the continuous variation of virus strains, the existing vaccine may exhibit reduced effectiveness. In addition, elderly patients may have difficulty swallowing the drug or poor compliance. Therefore, in addition to advocating early intervention, individual treatment should be focused on along with the actual condition of elderly patients. A combination of vaccine prevention and drug treatment should be considered for disease prevention and treatment in these patients.

Certain limitations of the present retrospective study included its small sample size and that drug accessibility was affected during the study. Therefore, the patients enrolled for treatment presented a more severe clinical baseline status compared to the currently approved indications. Nevertheless, the present study revealed that early use of BRII-196/198 resulted in more clinical end-point benefits. The consideration of variable factors, such as the combination of drugs, may also not have been adequately comprehensive in the present study. For instance, thrombocytopenia could occur due to the combined use of heparin and sodium in patients. Another limitation is that the study was conducted during the epidemic phases of delta virus spread, and the current Omicron strain, on which the risk of severe disease was tested, is weaker compared to the previous mutant strains. Therefore, a new mutant strain might escape certain neutralizing antibodies. However, recent preclinical data using live virus indicated that BRII-196/198 maintained its anti-viral activity against Omicron BA.2, BA.2.12.1, and BA.4/5[38]. Whether this conclusion is extendable to the current situation remains unclear so far, warranting further clinical exploration and verification.

## Conclusions

The timing of BRII-196/198 administration plays a great role in the treatment of COVID-19 for elderly patients aged over 60 years. The administration of BRII-196/198 within 3 days of the onset of symptoms may more significantly reduce the requirement for the use of high-flow oxygen or higher respiratory support in elderly patients aged over 60 years, which has certain guiding significance for the clinical application of neutralizing antibodies. Neutralizing antibody therapy should, therefore, be initiated as soon as possible for elderly mild or moderate COVID-19 patients over 60 years of age who do not require oxygen support, after a brief assessment of SpO2, and have risk factors for progression to severe COVID-19 disease. This may significantly reduce the worsening of disease progression. That is to say, for the patients who fulfill the guideline recommendations and drug indications, intervention of monoclonal neutralizing antibody therapy should be initiated at the early stage of COVID-19 disease.

## Data Availability

Available upon request.
